# Quantitative analysis of mitochondrial calcium uniporter (MCU) and essential MCU regulator (EMRE) in mitochondria from mouse tissues and HeLa cells

**DOI:** 10.1002/2211-5463.13371

**Published:** 2022-02-23

**Authors:** Akira Watanabe, Kousuke Maeda, Atsushi Nara, Mei Hashida, Mizune Ozono, Ayaka Nakao, Akiko Yamada, Yasuo Shinohara, Takenori Yamamoto

**Affiliations:** ^1^ 26360 Division of Molecular Target and Gene Therapy Products National Institute of Health Sciences Kawasaki‐shi Japan; ^2^ Institute for Genome Research Tokushima University Japan; ^3^ Faculty of Pharmaceutical Sciences Tokushima University Japan; ^4^ School of Dentistry Nihon University Chiyoda‐ku Japan

**Keywords:** calcium uniporter, EMRE, ion channel, MCU, mitochondria, stoichiometry

## Abstract

Mitochondrial calcium homeostasis plays critical roles in cell survival and aerobic metabolism in eukaryotes. The calcium uniporter is a highly selective calcium ion channel consisting of several subunits. Mitochondrial calcium uniporter (MCU) and essential MCU regulator (EMRE) are core subunits of the calcium uniporter required for calcium uptake activity in the mitochondria. Recent 3D structure analysis of the MCU‐EMRE complex reconstituted in nanodiscs revealed that the human MCU exists as a tetramer forming a channel pore, with EMRE bound to each MCU at a 1 : 1 ratio. However, the stoichiometry of MCU and EMRE in the mitochondria has not yet been investigated. We here quantitatively examined the protein levels of MCU and EMRE in the mitochondria from mouse tissues by using characterized antibodies and standard proteins. Unexpectedly, the number of EMRE molecules was lower than that of MCU; moreover, the ratios between MCU and EMRE were significantly different among tissues. Statistical calculations based on our findings suggest that a MCU tetramer binding to 4 EMREs may exist, but at low levels in the mitochondrial inner membrane. In brain mitochondria, the majority of MCU tetramers bind to 2 EMREs; in mitochondria in liver, kidney, and heart, MCU tetramers bind to 1 EMRE; and in kidney and heart, almost half of MCU tetramers bound to no EMRE. We propose here a novel stoichiometric model of the MCU‐EMRE complex in mitochondria.

## Graphical abstract

The calcium uniporter in the mitochondria is a highly selective calcium ion channel consisting of several subunits. In this study, we quantitatively examined the protein levels of core subunits of the calcium uniporter (MCU and EMRE). Unexpectedly, there were fewer EMRE molecules than MCU molecules; moreover, the MCU and EMRE ratios were significantly different among tissues.

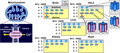

**Abbreviations**
AB: antibody
CBB: Coomassie brilliant blue
EM: electron microscopy
EMRE: essential MCU regulator
MCU: mitochondrial calcium uniporter
MCUR1: mitochondrial calcium uniporter regulator 1
MICU1: mitochondrial calcium uptake protein 1
MTS: mitochondrial targeting signal

Mitochondrial calcium homeostasis is crucial for cell survival and aerobic metabolism in eukaryotes [1, 2, 3]. However, excessive Ca^2+^ accumulation in mitochondria permeabilizes their inner membrane [4]. This phenomenon, referred to as permeability transition, results in cell death via processes such as apoptosis and necrosis [5, 6, 7], leading to different diseases [8, 9]. The mitochondrial calcium uniporter (MCU), located in the mitochondrial inner membrane, is a highly selective ion channel for Ca^2+^ uptake from the intermembrane space into the mitochondrial matrix [4, 10, 11]. In this decade, the molecular composition of the calcium uniporter complex was uncovered and found to consist of the MCU [12, 13]; mitochondrial calcium uptake proteins 1, 2, and 3 (mitochondrial calcium uptake protein 1 (MICU1), MICU2, and MICU3) [14, 15]; mitochondrial calcium uniporter regulator 1 (MCUR1) [16]; an MCU isoform (MCUb) [17]; and essential MCU regulator (EMRE) [18]. The MCU oligomer forms a channel pore [12, 13, 17]. MICU isoforms function cooperatively as regulatory factors of Ca^2+^ uptake by the MCU [3, 19, 20]; in addition, MICU1 is also related to Ca^2+^ selectivity [21]. MCUR1 has been reported to contribute to the stability of the MCU‐EMRE oligomer and to control the Ca^2+^ threshold [22, 23]. EMRE is essential for MCU‐dependent Ca^2+^ uptake, at least in mammalian mitochondria [18, 24, 25]; however, the reasons for its importance are not yet sufficiently understood.

Although the calcium uniporter is conserved in humans to worms, yeast lack mitochondrial calcium uptake activity. In a previous study, various subunits of mammalian calcium uniporter were expressed in yeast mitochondria [25]. As a result, although the expression of each subunit alone did not affect the mitochondrial calcium uptake activity, the co‐expression of MCU and EMRE enabled reconstitution of calcium uptake activity in yeast mitochondria [25]. This finding indicated that MCU and EMRE are key factors for the calcium uptake activity in mitochondria. To reveal the molecular mechanisms for the highly selective calcium transport, some independent groups analyzed the 3D structure of fungus MCUs [26, 27, 28, 29]. In the structures revealed from these studies, the MCU tetramer formed a channel pore, and 4 DxxE motifs in the MCU tetramer formed an ion‐selective filter at the pore entrance. Moreover, Wang et al. recently expressed human MCU and EMRE in insect cells; and a mixture of these recombinant proteins were functionally reconstituted on lipid‐bilayer nanodiscs [30]. The analysis of these discs by cryo‐electron microscopy revealed that, like fungus MCUs, human MCU was also observed as a channel pore‐forming tetramer, with EMRE bound to each MCU at a 1 : 1 ratio. In the MCU‐EMRE complex structure, the amino‐terminal regions of EMREs, which projected in matrix side, fixed the gate regions of the MCU tetramer in the open state. This 4 : 4 model of MCU‐EMRE complex also corresponded to the previous biochemical findings about MCU and EMRE obtained by topology analysis and mutation studies [17, 25, 31, 32].

In ion channels, the stoichiometry of subunits forming an ion channel often lead to changes in the properties of the ion channel. For example, in the Kv4.2 potassium ion channel in the plasma membrane, the number of KChIP subunits binding to a Kv4.2 tetramer affects the properties of potassium ion transport: the stoichiometry of Kv4.2 and KChIP affects the recovery rate from inactivated ion channels [33]. Based on such a report, the stoichiometry of MCU and EMRE could possibly be related to the activity and the properties of mitochondrial calcium uptake. However, the stoichiometry of MCU and EMRE in the mitochondria has not yet been investigated.

In this study, we established a quantitative analysis system of MCU and EMRE in mitochondria isolated from various mouse tissues and cultured cells by using antibodies and standard proteins. Using this system, we revealed the protein amounts of MCU and EMRE in various mitochondria and proposed a stoichiometry model of the MCU‐EMRE complex in the mitochondria. Finally, we discussed the relationships between our quantitative data and the previously reported mitochondrial calcium uptake activity.

## Materials and methods

### Materials

pET expression system 3 (code 69410‐3) was purchased from Novagen, Inc. (Madison, MI, USA); and maleimide‐activated keyhole limpet hemocyanin (code 77606), from Pierce Biotechnology, Inc. (Rockford, IL, USA). An ECL kit (code RPN2106) and anti‐rabbit IgG‐conjugated peroxidase (code NA934‐1ML) were obtained from GE Healthcare Life Science (Bucks, UK). Polyclonal antibody against human MCU (code HPA016480) was purchased from Sigma‐Aldrich Japan (Osaka, Japan). Polyclonal antibody against human EMRE (code sc‐86337) was obtained from Santa Cruz Biotechnology, Inc. (Dallas, TX, USA). HeLa cells were procured from the JCRB (Japanese Collection of Research Bioresources) Cell Bank. Dulbecco’s modified Eagle’s medium (code 05919) was purchased from Nissui (Tokyo, Japan).

### Preparation of mitochondria from mouse tissues and HeLa cells

Mitochondria were isolated from various tissues of 11‐week‐old male Slc:ddY mice, as described previously done with rat tissues [34]. The tissues were minced in +EDTA medium (250 mm sucrose, 2 mm Tris–HCl, 1 mm EDTA; pH 7.4) and then homogenized at low speed in a chilled Potter‐Elvehjem homogenizer. This homogenate was subsequently centrifuged for 5 min at 800 ***g*** at 4 °C to remove nuclei, erythrocytes, unbroken tissue cells, and debris, after which approximately three‐quarters of the supernatant was transferred to new tubes and centrifuged under the same conditions. The resulting supernatant was centrifuged for 10 min at about 6800 ***g***. The obtained crude mitochondrial pellet thus obtained was resuspended in +EDTA medium and centrifuged under the same conditions. The resulting pellet was resuspended and centrifuged for 10 min at 17,400 ***g***, and the subsequent pellet was resuspended and centrifuged under the same conditions except that EDTA medium (250 mm sucrose, 2 mm Tris–HCl, pH 7.4) was used.

Mitochondria were isolated from cultured HeLa cells in reference to Schmitt et al. [35]. HeLa cells (8 × 10^6^) were cultured in Dulbecco’s modified Eagle’s medium for 3 days. The cells were then washed in isolation medium (300 mm sucrose, 5 mm Tris–HCl, 200 µm EGTA; pH7.2). After centrifugation of the harvested cells for 5 min at 9000 ***g***, the pellet was resuspended and homogenized in a chilled Potter‐Elvehjem homogenizer. The homogenate was subsequently centrifuged for 10 min at 800 ***g***. The supernatant was transferred to new tubes and centrifuged for 10 min at 9000 ***g***, and the subsequent pellet was resuspended in isolation medium.

Mitochondrial protein concentrations were determined by performing the bicinchoninic acid assay with bovine serum albumin used as a standard.

### Generation of MCU knockout cells and EMRE knockout cells by the CRISPR/Cas9 method

MCU knockout cells and EMRE knockout cells were generated by using the CRISPR/Cas9 method reported previously [36]. Briefly, each single guide RNA (sgRNA) was designed to target the human MCU gene within its exon3 and the human EMRE gene within its exon 1. Specifically, the nucleotide sequences for these sgRNAs were as follows: MCU‐sgRNA, 5′‐TCCCGGCGTGAACGCTGTCA; EMRE‐sgRNA, 5′‐GGCTAGTATTGGCACCCGTC.

These sequences were cloned into a sgRNA vector, pSpCas9(BB)‐2A‐Puro (Addgene, #48139). HeLa cells were transfected with these plasmids, and the cells were incubated in medium containing 2 µg·µL^−1^ puromycin. After that, by picking up single colonies, the knockout clones were obtained. The cells were genotyped by using PCR and DNA sequencing.

### Preparation of expression vectors and yeast transformants

To prepare the expression vectors, we used pYO326/*TDH*p (*URA3* [or *LEU2*]), which is a multicopy vector with a glyceraldehyde‐3‐phosphate dehydrogenase (*TDH*) promoter and *URA3* (or *LEU2*) as a selection marker gene [25]. Mouse EMRE (Uniprot ID: Q9DB10) and the codon‐optimized mouse MCU, which was synthesized as described in our previous report [25], were used as templates of EMRE and MCU. MYC‐tagged EMRE and FLAG‐tagged MCU, both tagged at their C terminus, were inserted into the NdeI site and BamHI site of pYO326/*TDH*p (*URA3*) and pYO326/*TDH*p (*LEU2*), respectively. Deletion mutants of EMRE were prepared by PCR using each primer set shown in Table S1. Wild‐type yeast were transformed by the plasmids by using the lithium acetate transformation method. The yeast cells were incubated in SD medium lacking uracil or leucine for yeasts transformed with pYO326/*TDH*p(*URA3*) or pYO326/*TDH*p(*LEU2*), respectively.

### Preparation of antibodies

**Fig. 1:**
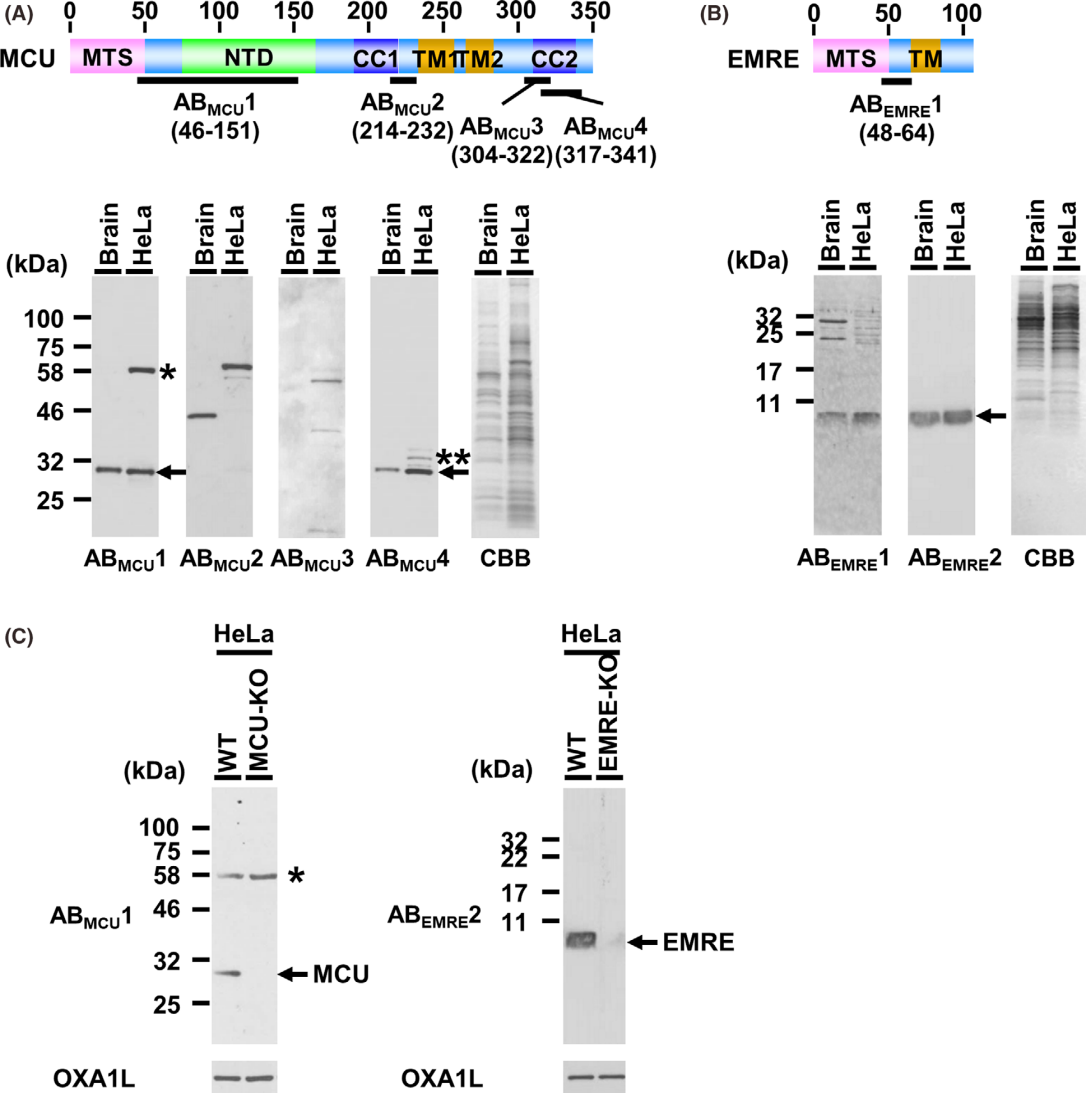
Evaluation of immunoreactivities of antibodies against MCU and EMRE. (A), Upper: Domains/motifs of mouse MCU. MTS, mitochondrial transit sequence (Met1‐Thr49); NTD, N‐terminal domain (Val74‐Arg164); CC, coiled‐coil domain (Ile191‐Arg220 for CC1, Arg310‐Gln338 for CC2); TM, transmembrane region (Leu233‐Glu256 for TM1, Val265‐Val282 for TM2). Schematic representation of epitopes of antibodies against mouse MCU; AB_MCU_1, AB_MCU_2, AB_MCU_3, AB_MCU_4: the location of each epitope is shown by the bold line. Lower: the mitochondria isolated from mouse brain and HeLa cells (5.0 and 10.0 µg·lane^−1^, respectively) were subjected to SDS/PAGE followed by CBB staining and immunoblotting using each MCU antibody. Mature MCU is shown by arrows; the bands formed by nonspecific reactions of the antibodies are indicated by asterisks. (B), Upper: Domains/motifs of mouse EMRE. MTS, mitochondrial transit sequence (Met1‐Ser47); TM, transmembrane region (Phe65‐Ile84 for TM). Schematic representation of epitopes of antibodies against mouse EMRE; AB_EMRE_1 Lower: immunoblots using each EMRE antibody and CBB staining; mature EMRE is shown by the arrow. (C), Immunoblot of the mitochondria isolated from wild‐type (WT) HeLa cells, MCU‐knockout HeLa cells, and EMRE‐knockout HeLa cells (10.0 µg·lane^−1^ of each), obtained by using AB_MCU_1 and AB_EMRE_2. As a loading control, human OXA1L was detected with anti‐OXA1L antibody (Santa Cruz, sc‐136011).

Polyclonal specific antibodies against mouse MCU (Uniprot ID: Q3UMR5) and mouse EMRE (Uniprot ID: Q9DB10) were prepared as described previously [37]. Antibodies against mouse MCU and EMRE were also raised by injection of synthetic peptides into adult New Zealand white rabbits. Amino acid sequences of these peptides used as immunogens were APLEKVRIEISRKAEKRTT (amino acids 214‐232 of mouse MCU, Fig. 1A), KGAKKSRFDLEKYNQLKDA (amino acids 304‐322 of mouse MCU, Fig. 1A), and NQLKDAIAQAEMDLKRLRDPLQVHL (amino acids 317‐341 of mouse MCU, Fig. 1A). These peptides were conjugated to keyhole limpet hemocyanin, then emulsified with Freund’s adjuvant, and injected into rabbits. Whole blood was obtained 10 days after the final booster shot and was allowed to stand at room temperature for 1 h and then overnight at 4 °C. The blood clot was removed by centrifugation at 5000 ***g*** for 10 min at 4 °C, and the resulting supernatant was used as an antiserum without further purification.

Polyclonal specific antibody against mouse EMRE was prepared by the same method as used for the mouse MCU. Amino acid sequence of the injected synthetic peptide used as the immunogen was VIVTRSGAILPKPVKMS (amino acids 48‐64 of mouse EMRE, Fig. 1B).

### Preparation of recombinant proteins of MCU

Codon‐optimized mouse MCU, which was synthesized as described in our previous report [25], was used as the template for the recombinant protein Mature‐form mouse MCU (His51‐Glu350) was amplified by PCR using forward primer 5′‐ AAAAAAACATATGCACCAGAGGCCGGCTTCCTG and reverse primer 5′‐GTAGGATCCTCATTCCTTTTCTCCGATC. The MCU cDNA was inserted into the NdeI site and BamHI site of pET‐3a. Escherichia coli BL21(DE3) pLysS cells were transformed with the obtained plasmids. For expression of MCU protein, *E. coli* transformants were incubated in LB medium containing ampicillin and chloramphenicol at 37 °C until the OD_600_ of the medium had reached 0.5. Isopropyl thio‐β‐D‐galactoside (0.4 mm) was then added to induce expression of the recombinant protein, after which incubation was continued at 22 °C for 4 h. Then, the cells were harvested by centrifugation followed by resuspension of the cell pellet in 10 mm Tris–HCl (pH 8.0) containing 1 mm EDTA (pH 7.8, TE), to which was added 10 mm dithiothreitol and 4% Triton X‐100. The cell suspension was freeze‐thawed and sonicated. After centrifugation, the pellet containing the inclusion bodies was resuspended in TE (crude MCU protein).

### Purification of recombinant MCU protein

The prepared crude MCU protein was resolved in 8 M guanidinium chloride. The proteins in the solution were separated by use of a reverse‐phase protein column (cytiva, RESOURCE RPC 3 mL) of an HPLC system (Shimadzu LC10‐AD) with elution using an acetonitrile gradient (5–40%). The obtained protein fractions containing recombinant MCU were subjected to freeze drying, and the protein powder was used as purified MCU in this study. The purified MCU powder was resolved in 1% SDS solution, and the concentrations of the MCU protein solution were determined by performing the bicinchoninic acid assay with bovine serum albumin used as a standard. The determination of the protein concentration was repeated 3 times, and the average of the obtained concentrations was used as the protein concentration of the MCU standard protein solution in this study.

### Chemical synthesis of EMRE protein

The chemical synthesis of mouse mature EMRE (Ser53‐Asp107) and the purification of the synthesized protein were carried out by the Sapporo Laboratory of Cosmo Bio Co., Ltd. (Tokyo, Japan). Briefly, the Ser53‐Asp107 sequence was synthesized by the Fmoc solid‐phase peptide synthesis method using Liberty Blue (CEM); and the synthesized product was purified by reverse‐phase protein column chromatography. The monoisotopic mass of the purified protein was determined by mass spectrometry (Shimadzu AXIMA Confidence). The observed monoisotopic mass was 6111.28 (theoretical monoisotopic mass, 6109.04). The purified EMRE pellet was resolved in 2% SDS solution, and the concentrations of the EMRE protein solution were determined by the same procedure as that used for the MCU protein solution.

### Protein detection by western blotting

Proteins obtained as described above were solubilized in extraction buffer (12.5 mm Tris–HCl, pH 6.8, containing 1% SDS, 10% glycerol, 1% dithiothreitol, and 0.05% bromophenol blue). SDS/PAGE was performed in 10% and 20% acrylamide gels essentially as described previously [38]. After transfer of the proteins in the gel to nitrocellulose membranes, the membranes were soaked for 1 h in TS buffer (20 mm sodium phosphate buffer, pH 7.4, containing 0.05% Tween 20, 150 mm NaCl, and skim milk (0.3% skim milk for anti‐MCU Ab1, AB_MCU_1; 1.0% for AB_MCU_2‐4: and 3.0% for AB_EMRE_1, 0.1% for AB_EMRE_2). The blocked membranes were then incubated overnight with antibodies against each peptide derived from mouse MCU or mouse EMRE with Can Get Signal (TOYOBO) as the diluent (AB_MCU_1‐3, 4000‐fold diluted; AB_MCU_4, 10000‐fold diluted; AB_EMRE_1, 2000‐fold diluted; AB_EMRE_2, 1000‐fold diluted). After having been washed with TS buffer, the membranes were incubated for 1 h with anti‐rabbit IgG conjugated with peroxidase as secondary antibody: AB_MCU_1‐3, 2000‐fold diluted; AB_MCU_4, 3000‐fold diluted; AB_EMRE_1‐2, 2000‐fold diluted with TS buffer). Specific binding of antibodies was visualized by the use of ECL reagents and subsequent exposure to x‐ray film (Fuji Photo Film, Osaka, Japan).

Determination of the amount of MCU and EMRE was performed as follows: First, to make a calibration curve, we subjected different but known amounts of MCU or EMRE to SDS/PAGE and then transferred them to a nitrocellulose membrane. Intensities of the immunodetected protein band of them were determined by using imagej, NIH, Bethesda, MD, USA.

### Calculation of the probability of the MCU‐EMRE complex with each stoichiometry

The probability of the MCU‐EMRE complex with each stoichiometry was obtained based on the ratio of MCU and EMRE in the mitochondria by fitting with a binomial distribution according to previous reports [33]. In the case that EMRE randomly associated with the MCU tetramer, the probability of the MCU‐EMRE complex with each stoichiometry was calculated by the following formula:

$$P\left(k\right){=}_{n}C_{k}\cdot p^{k}\cdot {\left(1‐p\right)}^{n‐k}$$

where *P*(*k*) represents the probability of the MCU‐EMRE complex with each stoichiometry, *n* represents the possible number of EMREs associated with a single MCU tetramer (*n* = 4); *k* represents the number of EMREs associated with a single MCU tetramer (*k* = 0–4); and p represents the molecular ratio of EMRE to MCU in the mitochondria (0 ≦ *P* ≦ 1).

## Results

### Characterization of antibodies used for the quantitative analysis of MCU and EMRE

For the quantitative analysis of MCU and EMRE, highly specific antibodies against each protein were necessary. Thus, we first originally prepared antibodies against MCU and EMRE, and we examined the immunoreactivity of these prepared antibodies and commercially available antibodies.

The commercially available antibody against human MCU, which we here used, was prepared by use of the recombinant protein of the N‐terminal region of human MCU (His47‐Val152) as an immunogen (shown as AB_MCU_1 in this study; Fig. 1A). Additionally, we synthesized 3 peptides corresponding to Ala214‐Thr232, Lys304‐Ala322, Asn317‐Leu341 of mouse MCU; and by use of these peptides as immunogens, we prepared their antisera (show as AB_MCU_2, AB_MCU_3, AB_MCU_4 [Fig. 1A]). To examine the specificity of the antibodies, we carried out western blotting of the mitochondria isolated from mouse brain and HeLa cells by using these 4 MCU antibodies. The identity of the protein sequences between moue MCU and human MCU was almost 100% (Fig. S1), allowing the prediction that these antibodies would show immunocrossreactivity to human MCU and mouse MCU. Consequently, in the case using AB_MCU_1 and AB_MCU_4, immunoreactive bands were clearly detected, showing migration corresponding to the predicted molecular weight of mouse and human mature MCU, that is, 34843.02 and 35027.34, respectively (see the arrows in Fig. 1A, lower panel). AB_MCU_2 and AB_MCU_3 did not immuno‐react with MCUs, the reasons being unclear. In the case using AB_MCU_1, a probably nonspecific immunoreactive band of high migration was detected (see the single asterisk in Fig. 1A, lower panel); while, in the case using AB_MCU_4, such a band was detected at a slightly higher migration than that of mature MCU (see the double asterisk in Fig. 1A, lower panel). From this result, we considered that AB_MCU_1 was more proper for the quantitative analysis of the immunoreactive band of MCU. For further investigation of the specificity of AB_MCU_1, we confirmed the immunoreactivity in the mitochondria isolated from MCU‐deficient HeLa cells. As a result, the immunoreactive band of mature MCU at around 30 kDa had completely disappeared. Meanwhile, the band detected at the higher migration was not affected, meaning that the higher migration band did not include MCU oligomer but other proteins nonspecifically reactive with AB_MCU_1 (Fig. 1C). These results showed that we could carry out quantitative analysis of MCU by using the signal intensities of the immunoreactive band migrating at about 30 kDa as detected by Ab_MCU_1.

**Fig. 2:**
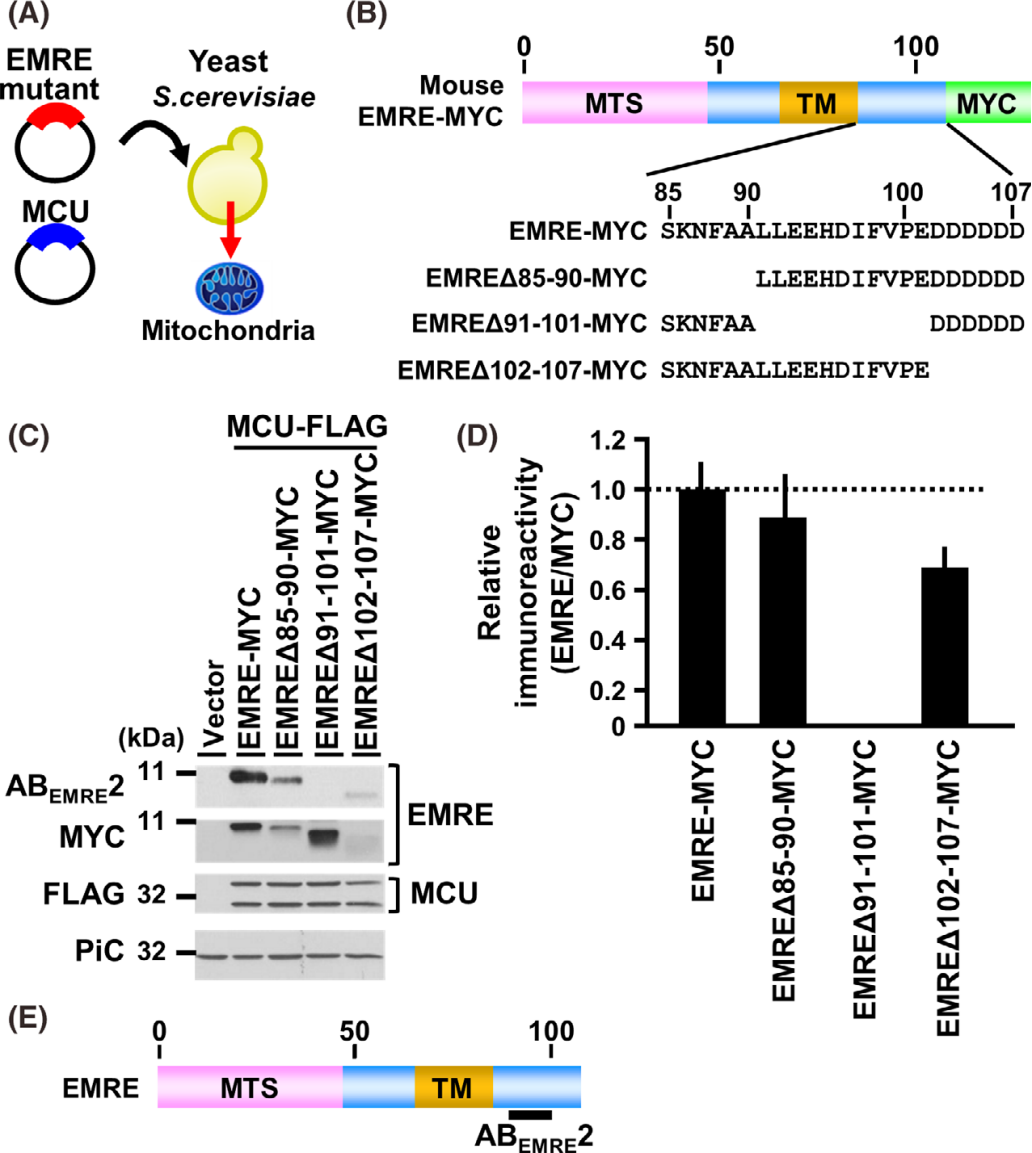
Identification of epitope regions of AB_EMRE_2 in mouse EMRE. (A), the yeast expression system of mouse EMRE and MCU enables reconstitution of the Ca^2+^ uptake function in yeast mitochondria. (B), Schematic representation of full‐length EMRE and its deletion mutants. (C), Immunoblots of mitochondria isolated from yeast expressing the above‐indicated MYC‐tagged EMRE deletion mutants together with FLAG‐tagged MCU, detected with AB_EMRE_2, anti‐MYC antibody for EMRE; anti‐FLAG antibody for MCU [upper and lower bands were immature MCU and mature MCU, respectively ([25])]. The mitochondrial phosphate carrier (PiC) was used as a loading control for the mitochondria. (D), Relative immunoreactivity of AB_EMRE_2 against each deletion mutant of EMRE. Each signal intensity detected by AB_EMRE_2 was divided by that detected by anti‐MYC antibody. The relative quotients are shown as histograms (mean ± SD); *n* ≥ 3. (E), Schematic representation of epitopes of antibodies against AB_EMRE_2.

Regarding EMRE, we prepared antibody AB_EMRE_1 by using the synthetic peptide corresponding to Val48‐Ser64 in the N‐terminal region of mature mouse EMRE as an immunogen. (Fig. 1B, upper panel). Some antibodies against EMRE are commercially available, for example, anti‐EMRE polyclonal antibody C22orf32(C‐12) previously used by some researchers (shown as AB_EMRE_2 in this study) [18, 31, 39, 40]. However, the epitope region of AB_EMRE_2 was unclear; so we examined the epitope region. For this, we first tried to prepare EMRE mutant proteins by using *E. coli*, but EMRE was hardly expressed in these bacteria (data not shown). We next used a yeast expression system; because, although yeast mitochondria lack the calcium uniporter complex, the expression of mammalian MCU and EMRE in yeast enables reconstitution of the calcium uptake function in yeast mitochondria [25]. For this, we expressed each deletion mutant of mouse EMRE in yeast; and not only EMRE but also MCU was expressed for EMRE’s stability in the mitochondria (Fig. 2A). The mitochondria isolated from the yeast strains were subjected to western blotting to determine the reactivity of AB_EMRE_2 against the various deletion mutants of mouse EMRE in yeast mitochondria (Fig. 2B). Since AB_EMRE_2 did not react with the EMRE mutant bearing the Leu91‐Glu101 deletion (EMRE∆91‐101), we concluded that the epitope region of AB_EMRE_2 mainly resided in the Leu91‐Glu01 sequence of mouse EMRE (Fig. 2C–E). Amino acid sequences of both epitopes completely corresponded to not only mouse EMRE but also the human one (Fig. S2). The reactivities of AB_EMRE_1 and AB_EMRE_2 toward the mitochondria isolated from mouse tissues and HeLa cells were examined. In the case of both antibodies, the immunoreactive bands were clearly detected at the migration corresponding to the molecular weight of mature mouse EMRE, that is, 6109.04 (Fig. 1B). AB_EMRE_2 detected a single band, while AB_EMRE_1 nonspecifically reacted with some proteins having a higher molecular weight, meaning that AB_EMRE_2 was better than AB_EMRE_1 for the detection of EMRE in isolated mitochondria. Furthermore, when AB_EMRE_2 was reacted with mitochondria isolated from EMRE‐deficient HeLa cells, the immunoreactive band at 10 kDa was completely absent, indicating that AB_EMRE_2 would be useful for the quantitative analysis of EMRE (Fig. 1C).

From these result, we used AB_MCU_1 and AB_EMRE_2 for the detection of MCU and EMRE, respectively, in the following experiments.

### Preparation of recombinant MCU and EMRE as standard proteins

**Fig. 3:**
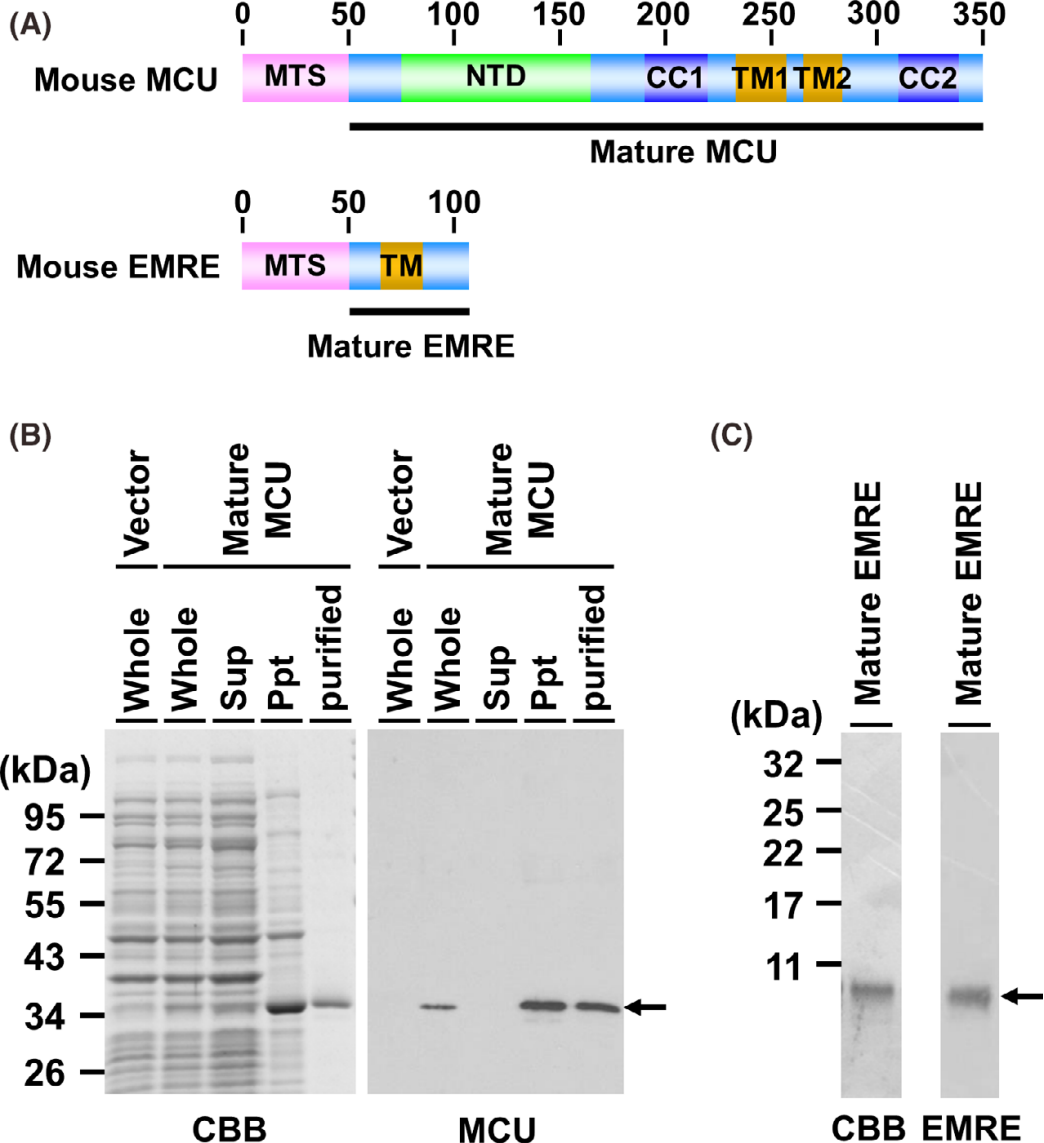
Preparation of mature MCU and EMRE as standard proteins. (A), Schematic representation of mouse MCU and EMRE: the locations of the mature MCU and EMRE are shown by the bold lines: Ala50‐Glu350 in mouse MCU and Ser53‐Asp107 in mouse EMRE. (B), the indicated protein samples were subjected to SDS/PAGE followed by CBB staining (Left) and western blotting using AB_MCU_1 (Right): whole cell lysate of *E. coli* transfected with empty vector: that transfected with expression vector of mouse mature MCU (Whole); the supernatant fraction (Sup) and the precipitate fraction (Ppt) after centrifugation of the whole cell lysate; the purified fraction of mature MCU obtained by using reverse‐phase chromatography (Purified). (C) Chemically synthesized EMRE was subjected to SDS/PAGE followed by CBB staining and western blotting using AB_EMRE_2.

Met1‐Thr49 of mouse MCU functions as a mitochondrial targeting sequence (MTS); and it is truncated after the translocation of MCU in mitochondria, resulting in the mature form of MCU (Fig. 3A). When the mature MCU was expressed in *E. coli*, a high amount of the protein was obtained in the pellet after centrifugation of whole cell lysates, indicating that the expressed MCU existed in an inclusion body (Fig. 3B). Then, the crude MCU was purified by using reverse‐phase chromatography. When the purified MCU fraction was subjected to SDS/PAGE followed by Coomassie brilliant blue (CBB) staining, a clear single band was detected at the migration indicating the molecular weight of mature MCU, that is, 34843.02 (Fig. 3B, left); furthermore, AB_MCU_1 specifically reacted the bands (Fig. 3B, right). We also tried to establish the E. coli expression system of mouse mature EMRE. However, the expression level of EMRE protein was very poor; and so any examination of the conditions required to achieve a sufficiently high expression level of EMRE could not be performed (data not shown). Although the reason is unclear, the poor expression of EMRE in *E. coli* might be related to the highly hydrophobic structure of mature EMRE, as hydrophobic amino acid residues account for about 50% of the residues of mature EMRE. Since mature EMRE was a small protein, that is, M.w. 6109.04, we next attempted the chemical synthesis of it. Stefani et al. reported that human EMRE was truncated at Arg52 [39]. Therefore, we chemically synthesized Ser53‐Asp107 of mouse EMRE. When the synthesized matured EMRE was subjected to SDS/PAGE followed by CBB staining, the synthesized protein was detected as a single band; and no other protein bands were detected. Anti‐EMRE antibody also reacted with the protein band detected in the CBB‐stained gel (Fig. 3C, right). Furthermore, to roughly confirm the protein sequences, the prepared proteins of MCU and EMRE were digested by trypsin/Lys‐C and then subjected to LC‐MS/MS analysis, resulting that the peptides derived from mouse MCU and EMRE were identified (Fig. S3, Tables [Link], [Link]).

Since the recombinant proteins of MCU and EMRE having high purity were obtained, we used these proteins as standards for subsequent quantitative analysis.

### Quantitative analysis of MCU and EMRE in the mitochondria isolated from tissues and cultured cells

**Fig. 4:**
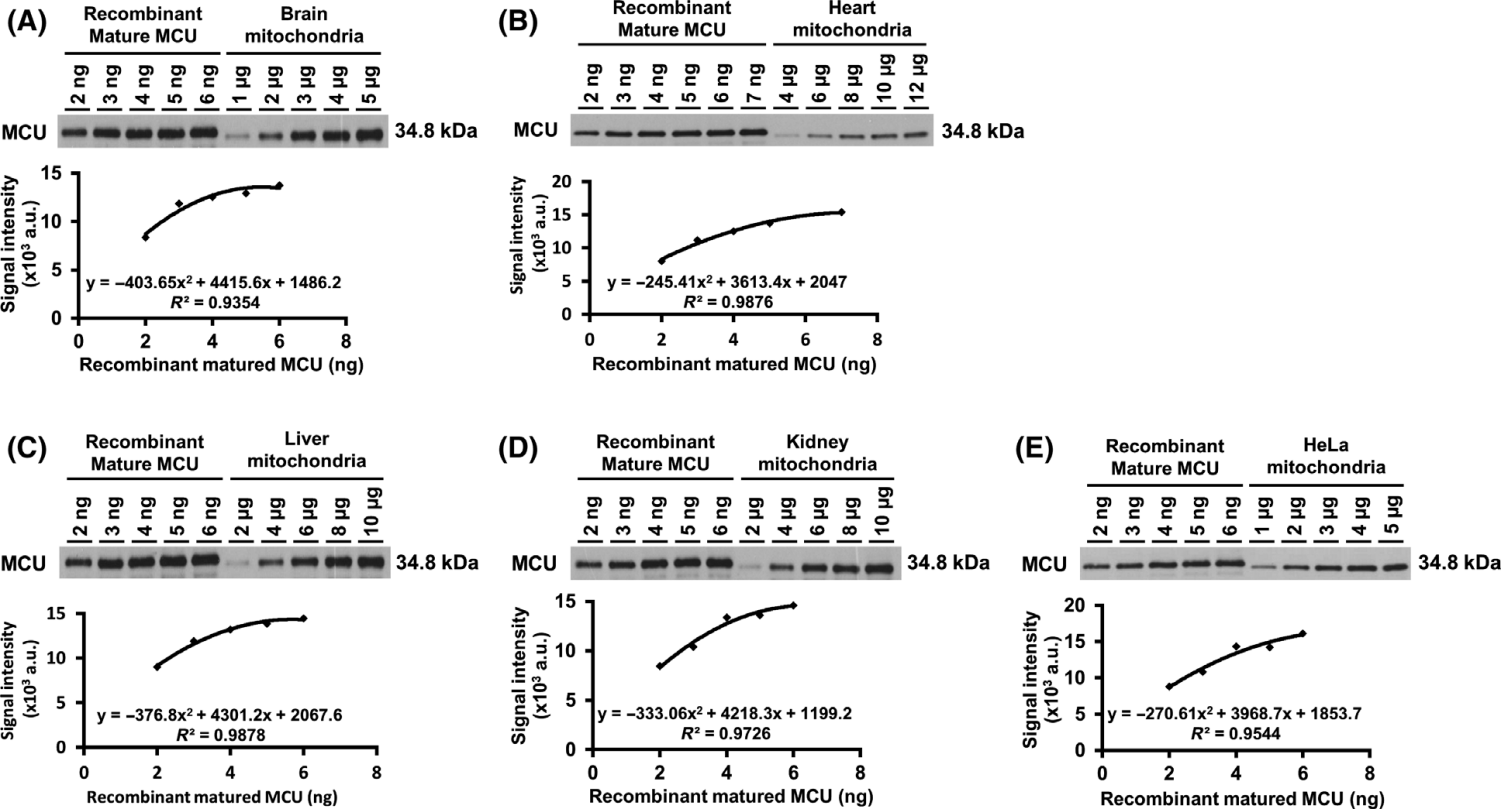
Quantification analysis of the expression levels of MCU in the mitochondria. Various amounts of the recombinant mature MCU as the standard protein and the mitochondria isolated from mouse brain (A), heart (B), liver (C), kidney (D), and HeLa cells (E) were subjected to immunoblotting using AB_MCU_1 antibody. The signal intensity of each band of recombinant mature MCU was detected by imagej; and those signal intensities were plotted, resulting in calibration curves. By use of the calibration curves, the amounts of MCU protein in the mitochondria were calculated from the signal intensities detected from each amount of the mitochondria; and the averages of the calculated mitochondrial amount are shown in Table 1 (mean ± SD); *n* ≥ 3.

**Fig. 5:**
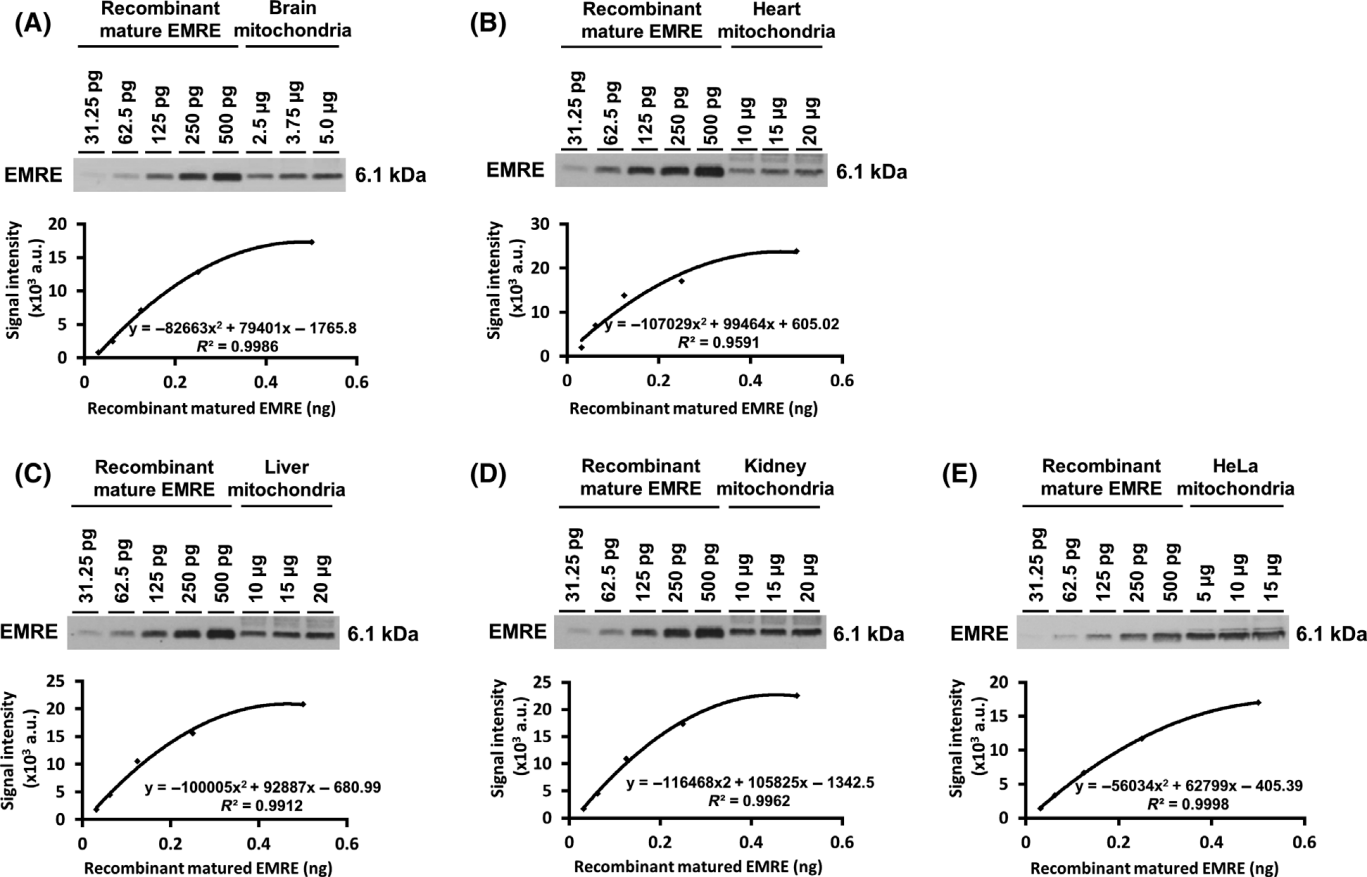
Quantification analysis of the expression levels of EMRE in the mitochondria. Various amounts of the recombinant mature EMRE as the standard protein and the mitochondria isolated from mouse brain (A), heart (B), liver (C), kidney (D), and HeLa cells (E) were subjected to immunoblotting using AB_EMRE_2 antibody. By the same procedure as in the case of MCU (Fig. 4), the protein amounts of EMRE in the mitochondria were calculated from the signal intensities detected from each amount of the mitochondria; the averages of the calculated mitochondrial amount are shown in Table 1 (mean ± SD); *n* ≥ 3.

To reveal the amount of MCU protein in the mitochondria of tissues and cultured cells, we subjected serial amounts of the recombinant MCU and the mitochondrial suspensions isolated from mouse brain, heart, liver, kidney, and HeLa cells to SDS/PAGE in the same gel followed by western blotting (Fig. 4). From the signal intensities in the lanes loaded with recombinant MCUs, calibration curves were obtained; and each correlation coefficient (*R* ^2^) was high. Using the calibration curve obtained from the serial recombinant MCU, we calculated the amount of MCU protein in the mitochondria of each tissue and HeLa cells. Based on the signal intensity detected in each lane loaded with each of the various mitochondrial sources, the amount of MCU per 1 μg of mitochondria from these various sources was calculated. After statistical processing using the Smirnov‐Grubbs test, the average among those MCU amounts per 1 μg was calculated, providing the content of MCU in the mitochondria from each tissue or HeLa cells (Table 1). The migration of MCU in tissues and HeLa cells detected by SDS/PAGE was slightly lower than that of the recombinant mature MCU, the reason being unclear. We had added Met at the N terminus of the recombinant mature MCU for its expression in *E. coli*. Since a single amino acid mutation in a protein often affects the migration of the protein during SDS/PAGE [37], this additional N‐terminal Met might possibly account for the different migration of the mature MCU. The N terminus of mature MCU in mitochondria has not yet been experimentally investigated; actually, the mitochondrial targeting sequence (MTS) of MCU might not be cleaved at Thr49 but at its downstream. Regarding the cleavage site of MTS, further studies will be needed. As in the case of MCU, the amounts of EMRE protein in the mitochondria were also examined (Fig. 5; Table 1).

**Table 1:** The expression levels of MCU and EMRE in the mitochondria of mouse tissues.

Tissue or cell	MCU		EMRE		MCU : EMRE^c^
	pg·µg^−1^ ^a^	fmol·µg^−1^ ^b^	pg·µg^−1^ ^a^	fmol·µg^−1^ ^b^	(mole ratio)
Brain	728.52 (±29.73)	20.91 (±0.85)	50.30 (±7.80)	8.23 (±1.28)	1.00 : 0.39 (±0.06)
Heart	229.47 (±45.88)	6.58(±1.32)	5.90 (±0.35)	0.97(±0.06)	1.00 : 0.15 (±0.04)
Liver	385.00 (±90.34)	11.05 (±2.59)	15.05 (±3.64)	2.46 (±0.60)	1.00 : 0.24 (±0.09)
Kidney	480.84 (±46.73)	13.8 (±1.34)	15.73 (±3.50)	2.58 (±0.57)	1.00 : 0.19 (±0.05)
HeLa	1000.08 (±88.56)	28.7 (±2.54)	53.16 (±2.30)	8.71 (±0.38)	1.00 : 0.31 (±0.03)

^a^ The amounts of MCU or EMRE protein in 1 µg of the mitochondria.

^b^ The number of moles of MCU or EMRE protein in 1 µg of the mitochondria.

^c^ The ratios of the number of moles of EMRE to that of MCU in the mitochondria.

**Fig. 6:**
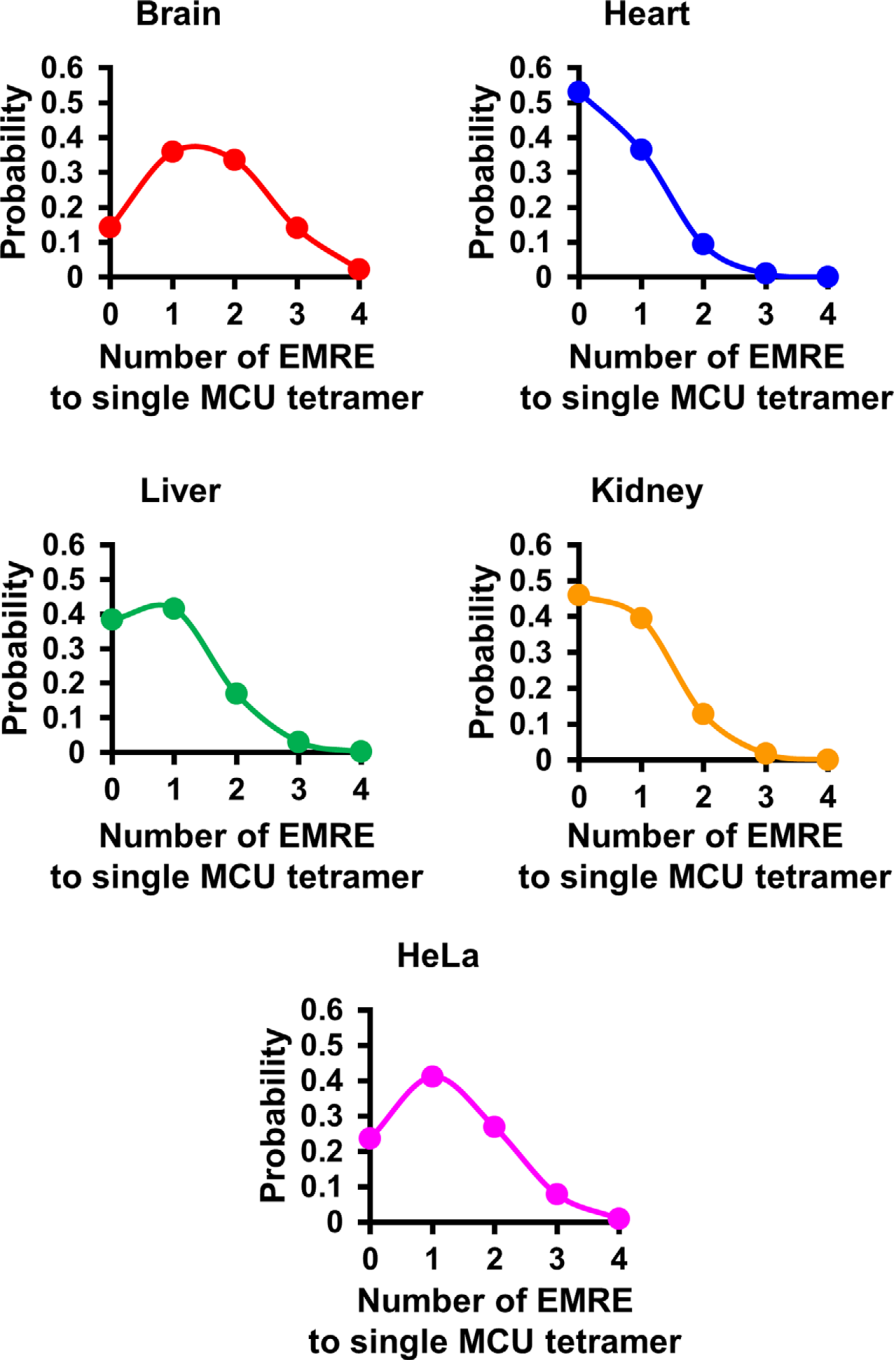
Probability description of expected number of EMRE to a MCU tetramer. Molecular ratios of EMRE to MCU in the mitochondria isolated from mouse tissues and HeLa cells, which were shown in Table 1, were used to determine the probability distribution function from the binomial theorem. From the calculation, the probability description of the expected number of EMRE (0–4) to a MCU tetramer was obtained and plotted in histograms.

**Fig. 7:**
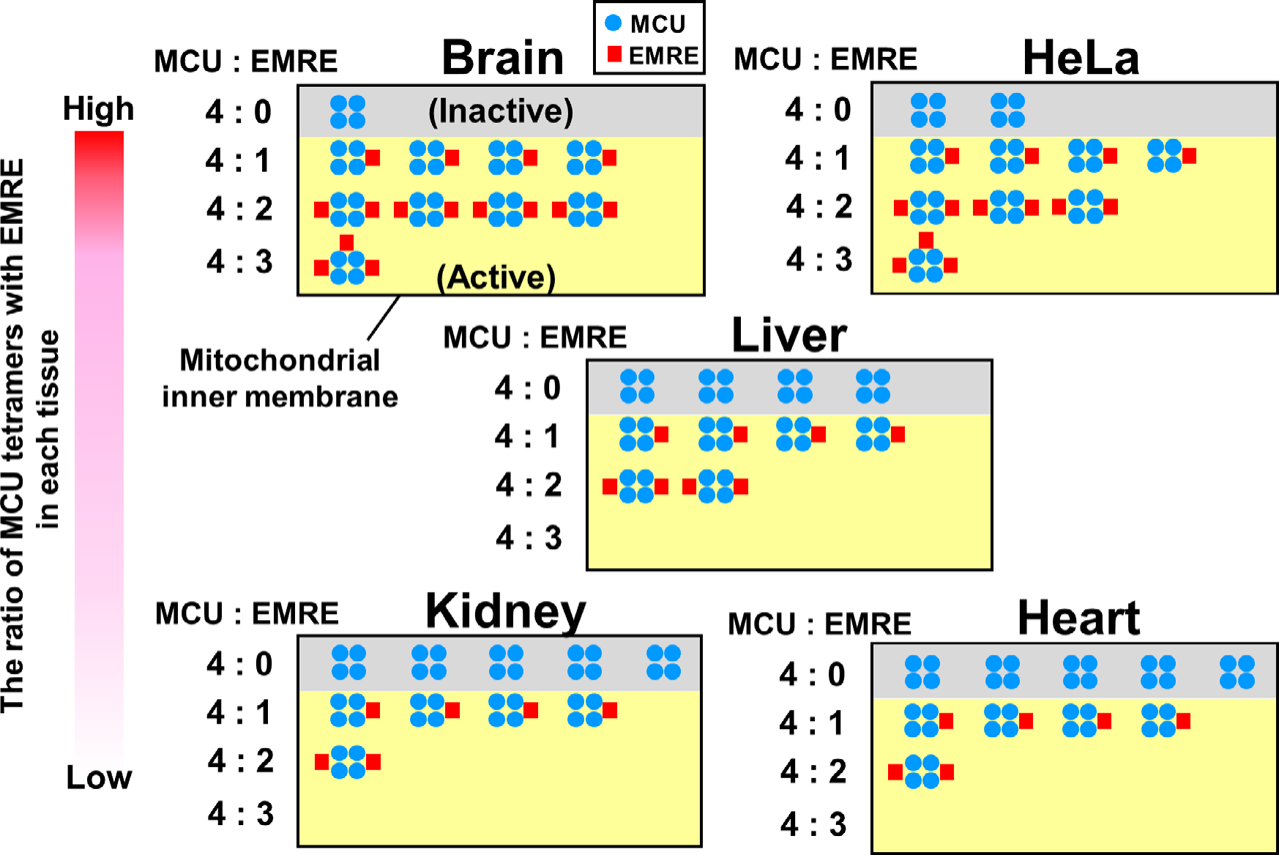
Variation and composition of MCU‐EMRE complexes in mitochondrial inner membranes based on the stoichiometry of MCU and EMRE. This study showed that, in all tissues and HeLa cells, 0‐3 molecules of EMRE were assembled in a single MCU tetramer. Based on the probabilities of each complex (MCU : EMRE = 4 : 0, 4 : 1, 4 : 2, 4 : 3), which were obtained in Fig. 6, the ratios of each MCU‐EMRE complex in the mitochondrial inner membrane were calculated. By use of these ratios, the breakdown of each complex in 10 MCU tetramers is illustrated. In this model, MCU tetramers without EMRE are shown as functionally inactive (grey). MCU tetramers with 1 EMRE, those with 2 EMREs, and those with 3 EMREs were assumed to be functionally active (yellow).

Based on the molecular weights of the mature forms of MCU and EMRE (34843.02 and 6109.04, respectively), the number of moles of each protein (fmol·μg^−1^ of mitochondria) was calculated, as were the ratios of the number of moles of EMRE to that of MCU in the mitochondria (Table 1). The molecular ratios of EMRE to MCU in the mitochondria isolated from mouse tissues and HeLa cells were used to determine the probability distribution function from the binomial theorem. From the calculation, probability description of the expected number of EMRE (0–4) to a MCU tetramer were obtained and plotted in histograms (Fig. 6). Based on the probabilities of each complex (MCU : EMRE = 4 : 0, 4 : 1, 4 : 2, 4 : 3), the ratios of each MCU‐EMRE complex in the mitochondrial inner membrane were calculated; and using these ratios, the breakdown of each complex in 10 MCU tetramers was illustrated (Fig. 7).

## Discussion

In this study, to reveal the amounts of MCU and EMRE proteins, the signal intensities of the recombinant MCU and the chemically synthesized EMRE were compared with those of MCU and EMRE in the mitochondria, respectively. In this analysis, it was necessary that the reactivity of each antibody to the recombinant protein as a standard was the same as that to the intrinsic protein. In the case that the sequences of epitopes had post‐translational modifications, the reactivity of the antibody could be different between the standard protein and the intrinsic one. Indeed, in the sequence of the epitope of AB_MCU_1, cleavage at Thr49 and the phosphorylation of Ser92 in human MCU were reported [37, 41, 42]. However, when the mitochondrial MCU amounts were examined by using another anti‐MCU antibody, AB_MCU_4, which we here prepared by using the peptide of Asn317‐Leu341 of mouse MCU as the immunogen, the obtained MCU amounts were almost the same as those obtained by AB_MCU_1 (Fig. S4). This finding indicated that the MCU amounts obtained from this study did not include errors caused by any difference in reactivity of the antibodies to intrinsic MCU and the recombinant one, meaning that this assay enabled correct measurement of the amount of MCU protein. On the other hand, EMRE is truncated at Arg52 after translocation to mitochondria [39]; and any other truncation sites have not yet been reported. When the amount of EMRE in the mitochondria was investigated by using antibody AB_EMRE_1, which was prepared by using the Val48‐Ser64 peptide, almost the same amount of EMRE as that obtained by AB_EMRE_2 (Fig. 5) was detected (Fig. S5), indicating that this assay afforded the correct amount of EMRE.

In the present study, we showed for the first time the amounts of MCU and EMRE proteins in the mitochondria. From this result, the protein amount (pg·μg^−1^) and the number of moles (fmol·μg^−1^) of MCU were found to be larger than those of EMRE in the mitochondria of each tissue and HeLa cells (Table 1). In the liver mitochondria, the amount of MCU protein was 385 pg per 1 μg of whole mitochondrial protein, indicating that the MCU occupied approximately 0.04%(w/w) of the whole protein weight of the liver mitochondria. The voltage‐dependent anion channel, which is an abundant protein in mitochondria, accounts for approximately 0.4%(w/w) [37]. These data show that the MCU is not an abundant protein, indicating that the amount of calcium uniporter complex is comparatively small. This finding is in line with an expectation that the amount of calcium uniporter in the mitochondria might be small, which is based on very low IC_50_ of Ru360, an inhibitor of the calcium uniporter [43]. In this study, the protein levels of MCU and EMRE in mitochondria isolated from HeLa cells were higher than those in mitochondria isolated from mouse tissues. In general, the purity of mitochondria isolated from cultured cells is lower than that of mitochondria isolated from tissues. Our findings indicate that MCU and EMRE are highly expressed in the mitochondria of HeLa cells. Some researchers observed that yeast mitochondria lack calcium transport activity [24, 25, 43]. Although the expression of mammalian MCU alone in yeast does not enable Ca^2+^ transport in the yeast mitochondria, the co‐expression of mammalian MCU and EMRE does [24, 25], indicating that both MCU and EMRE are essential factors for mitochondrial calcium transport. In other words, MCUs without EMREs (reversely, EMREs without MCUs) are inactive in Ca^2+^ transport. In this study, the amounts of EMRE in the mitochondria of all tissues and HeLa cells were clearly less than the amounts of MCU. This finding suggests that the amount of EMRE is the bottle neck to decide the amount of active calcium uniporter under physiological conditions and that the amount of EMRE possibly affects the potency of mitochondrial calcium uptake and its variation among tissues. Indeed, Kirichok et al. investigated the mitochondrial calcium uptake activity among mouse various tissues by using patch‐clamp techniques, with the results indicating that the calcium uptake activity in liver and kidney were higher than that in heart [44]. In this study, the amount of EMRE in liver and kidney was larger than that in the heart (Table 1); and so the relationship between the magnitude of the EMRE amount among tissues corresponded to that of the calcium uptake activities.

Very recently, by using cryo‐EM Wang et al. [30] reported the 3D structure of the complex of human MCU and EMRE reconstituted in nanodiscs. The stoichiometry of MCU and EMRE in the complex was 4 to 4: each MCU bound to an EMRE in the complex. Unexpectedly, we here revealed that the stoichiometry of MCU and EMRE in the mitochondria was not 4 to 4; instead, the number of moles (fmol·μg^−1^) of EMRE was approximately 2–7 times lower than that of MCU in the mitochondria of various tissues and HeLa cells (Table 1). What is the stoichiometry of MCU and EMRE in the calcium uniporter complex in the mitochondria? Regarding the Kv4.2 potassium ion channel, Kitazawa et al. investigated the stoichiometry of the pore‐forming Kv4.2 tetramer and its accessory protein, the K^+^‐interacting protein (KChIP) subunit [33]; consequently, although Kv4.2 tetramers bound by 1–4 KChIPs was found to randomly exist in Xenopus oocytes, the number of KChIP bound to each Kv4.2 tetramer increased in proportion to the expression level of KChIPs. Based on this finding, if EMRE stochastically bound to MCU tetramers in proportion to the expression level of EMRE, the MCU tetramer binding to 4 EMREs would hardly exist in the mitochondrial inner membrane; in brain, the MCU tetramer bound to 1 and 2 EMRE(s) would be the major one; in liver and HeLa cells, that binding to 1 EMRE would majorly exist: in kidney and heart, the MCU tetramer with 1 EMRE would be major, and almost half of MCU tetramers would be without EMRE (Figs 6 and 7). Recently, Payne et al. [45] reported that the MCU tetramer with 1 EMRE also retained Ca^2+^ uptake activity, based on a study using the fusion protein tandemly connecting 4 MCUs and 1 EMRE. This report supports the above‐described consideration that we here suggested: that the MCU tetramer with 1 EMRE was the main stoichiometry in the mitochondria. Our findings indicated that MCU channels binding to various numbers of EMRE(s) exist in the mitochondria, which suggests that EMRE might enable precise regulation of mitochondrial calcium uptake among tissues and cells by not only its expression level but also by its number binding to each MCU tetramer. More quantitative studies will be needed to clarify whether the number of EMREs binding to the MCU tetramer affects the Ca^2+^ uptake activity and its properties, for example, the threshold of the Ca^2+^ uptake. For this, the analysis of Ca^2+^ uptake activity in brain mitochondria might be useful, because our data indicated that the MCU tetramer with 2 EMREs would be the major form in brain, whereas, in other tissues, the MCU tetramers with 1 EMRE or without EMRE would be the majority (Figs 6 and 7).

The results of MCU and EMRE quantification in this study suggested that 0–2 EMREs were mainly bound to MCU tetramers in the mitochondria of various mouse tissues. In contrast, Fan et al. recently used baculoviruses to produce high expression levels of human MCU, EMRE, MICU1, and MICU2 (Wang et al. highly expressed human MCU, EMRE, and MICU1), after which they observed their three‐dimensional structures using cryo‐electron microscopy [30, 46]. Their results showed that MCU and EMRE were bound at a ratio of 4 : 4. The reason for the discrepancy between these results and ours is unknown. We analyzed mitochondria isolated from mouse tissues, whereas Fan et al. analyzed mitochondria under conditions in which each subunit was highly expressed in baculoviruses. The difference in the amount of each subunit may change the compositional ratio of the subunits that make up the MCU complex. To clarify this issue, further studies will be required. Furthermore, in the native PAGE results of Payne et al., [45] a 4 : 2 complex was detected in HEK293T, which led the authors to conclude that this form was required for MICU1 gatekeeping. Fan et al. [46] also suggested that at least two EMREs are required for MICU1 gatekeeping (i.e., for suppressing low concentrations of Ca^2+^). The present study focused only on the stoichiometric ratio of MCU and EMRE, but it is possible that this ratio could be affected by other subunits such as MICU1 and MICU2.

The stochastic calculation using the binomial theorem suggested that a large number of MCU tetramers without EMREs, which lack the Ca^2+^ uptake function [24, 25], would exist in the mitochondria of all tissues and HeLa (Fig. 7). The reasons why such a large number of inactive calcium uniporters would exist in the mitochondria is unclear. Liu et al. reported that the expression level of EMRE increased more strongly than that of MCU in LAMA2‐knockout mice, which show a muscular dystrophy‐like phenotype [47]; Chen et al. reported that the expression level of EMRE, but not that of MCU, increased in Bx‐PC3 cells overexpressing HINT2 [48]. These findings suggest that the mitochondrial Ca^2+^ uptake activity might be regulated by the expression level of EMRE in response to any stimuli and that to enable such a regulation, a certain amount of inactive calcium uniporter might be maintained under normal conditions.

In conclusion, we for the first time quantitatively compared the amounts of MCU and EMRE in mouse mitochondria and discovered that the stoichiometry of MCU and EMRE in the mitochondria widely varied among tissues and cells. This finding provides new insights into the mechanisms regulating mitochondrial calcium uptake.

## Conflict of interest

The authors declare that they have no conflicts of interest with the contents of this article.

## Author contributions

AW, AY, and TY conceived the experiments; AW, KM, AtN, MH, MO, and AyN performed the experiments; AW, AY, YS, and TY performed the data analysis; TY wrote and revised the manuscript. All authors reviewed the final manuscript.

## Supporting information


**Fig. S1.** Alignment of amino acid sequence of human MCU and mouse MCU; Q8NE86 and Q3UMR5, respectively. Asterisks show amino acids conserved between the 2 MCUs; and dots, similar amino acids. The epitopes of anti‐MCU antibodies are shown in red and by underbars.
**Fig. S2.** Alignment of amino acid sequence of human EMRE and mouse EMRE; Q9H4I9 and Q9DB10, respectively. Asterisks show amino acids conserved between the 2 EMREs; and dots, similar amino acids. The epitopes of anti‐EMRE antibodies are shown in blue and by underbars.
**Fig. S3.** Peptide mapping of the prepared proteins of MCU and EMRE. (A), The peptides identified by MS analysis were shown by black boxes in mouse MCU protein sequence: (B), the identified peptides were shown in EMRE protein sequences. The brief procedure of MS analysis is the followed. The prepared proteins of MCU and EMRE were purified by methanol/chloroform precipitation. The protein pellets were solubilized in 8 M urea followed by reduction in 2.5 mM DTT for 30 min at 37 °C, and alkylation in 55 mM iodoacetamide for 30 min at room temperature in the dark. After reducing the urea concentration to 1 M using 50 mM Tris‐HCl (pH 8.0), the proteins were digested at 37 °C overnight using 100 ng of trypsin/Lys‐C mix, Mass Spec Grade (Promega, Madison, WI). The prepared peptides were desalted with GL‐Tip SDB (GL Sciences, Tokyo, Japan), and the eluates were concentrated using a SpeedVac concentrator (Thermo Fisher Scientific). The prepared peptide solutions were dissolved with 0.1% TFA. LC‐MS/MS analysis of the prepared peptides was carried out on an EASY‐nLC 1200 UHPLC connected to a Q Exactive Plus mass spectrometer (Thermo Fisher Scientific). The peptides were separated on a 75‐μm inner diameter × 120‐mm C18 reversed‐phase column (Nikkyo Technos, Tokyo, Japan) using a linear gradient from 5 to 40% acetonitrile for 0‐60 min. A data‐dependent acquisition mode was used as the operation program of the mass spectrometer. Raw data were analyzed using the UniProt database of Mus musculus with Proteome Discoverer, version 2.2 (Thermo Fisher Scientific) for peptide identification. The detail of the identified peptide were shown in Supplemental Table SII for recombinant MCU and Supplemental Table SIII for synthesized EMRE.
**Fig. S4.** Quantification analysis of MCU in the mitochondria by using anti‐MCU antibody, AB_MCU_4. A, Schematic representation of epitopes of antibodies against mouse MCU; AB_MCU_1 and AB_MCU_4: the location of each epitope is shown by the bold line. B,C, upper, recombinant mature MCU protein (prepared in Figure 3) and the mitochondria isolated from mouse liver (B) and kidney (C) were subjected to SDS‐PAGE followed by immunoblotting using AB_MCU_4; lower, The signal intensity of each band of recombinant mature MCU was detected by Image J; those signal intensities were plotted, resulting in calibration curves. The calculation of the amount of MCU protein from the signal intensities was carried out as shown in the legend of Fig 4. This amount was obtained by dividing the amount of MCU protein by the molecular weight (34843.02) of the mature MCU. The amount of MCU (fmol/µg) of obtained by using AB_MCU_4 (shown in light blue in the histogram) was compared with that obtained with AB_MCU_1 (shown in blue histogram) and shows that the amounts of MCU in liver mitochondria and kidney were almost the same.
**Fig. S5.** Quantification analysis of EMRE in the mitochondria by using anti‐EMRE antibody, AB_EMRE_1. A, Schematic representation of epitopes of antibodies against mouse EMRE; AB_EMRE_1 and AB_EMRE_2: the location of each epitope is shown by the bold line. B, upper, the recombinant mature EMRE (prepared in Figure 3) and the mitochondria isolated from mouse liver were subjected to SDS‐PAGE followed by immunoblotting using AB_EMRE_1; lower, The signal intensity of each band of recombinant mature EMRE was detected by Image J; and those signal intensities were plotted, resulting in calibration curves. The calculation of the amount of EMRE protein from the signal intensities was carried out as shown in the legend of Fig 5. The averages of the calculated mitochondrial amount are shown in the histogram (C; mean ±s.d.), n≥3). C, The amount of EMRE (fmol) in 1 μg of the isolated mitochondria. This amount was obtained by dividing the amount of EMRE protein by the molecular weight (6109.04) of mature EMRE. The amount of EMRE (fmol/µg) obtained with AB_EMRE_1 (shown by the pink bar of the histogram) was compared with that using AB_EMRE_2 (shown by the red bar) and shows that the amounts of EMRE in liver mitochondria were almost the same with both antibodies.

**Table S1.** Primers used for preparation of expression vectors of the EMRE mutants.

**Table S2.** MS/MS analysis of the recombinant mouse MCU.

**Table S3.** MS/MS analysis of the synthesized mouse EMRE.

## Data Availability

All data in our study are available from the corresponding author on reasonable request.

## References

[1] McCormack JG , Denton RM . The role of mitochondrial Ca^2+^ transport and matrix Ca^2+^ in signal transduction in mammalian tissues. Biochim Biophys Acta. 1990;1018:287–91. 10.1016/0005-2728(90)90269-A 2203475

[2] Pizzo P , Drago I , Filadi R , Pozzan T . Mitochondrial Ca^2+^ homeostasis: mechanism, role, and tissue specificities. Pflugers Arch. 2012;464:3–17. 10.1007/s00424-012-1122-y 22706634

[3] Patron M , Checchetto V , Raffaello A , Teardo E , Vecellio Reane D , Mantoan M , et al. MICU1 and MICU2 finely tune the mitochondrial Ca^2+^ uniporter by exerting opposite effects on MCU activity. Mol Cell. 2014;53:726–37. 10.1016/j.molcel.2014.01.013 24560927 PMC3988891

[4] Gunter TE , Pfeiffer DR . Mechanisms by which mitochondria transport calcium. Am J Physiol. 1990;258:C755–86. 10.1152/ajpcell.1990.258.5.C755 2185657

[5] Bernardi P . Mitochondrial transport of cations: channels, exchangers, and permeability transition. Physiol Rev. 1999;79:1127–55. 10.1152/physrev.1999.79.4.1127 10508231

[6] Giorgio V , von Stockum S , Antoniel M , Fabbro A , Fogolari F , Forte M , et al. Dimers of mitochondrial ATP synthase form the permeability transition pore. Proc Natl Acad Sci USA. 2013;110:5887–92. 10.1073/pnas.1217823110 23530243 PMC3625323

[7] Giorgio V , Burchell V , Schiavone M , Bassot C , Minervini G , Petronilli V , et al. Ca^2+^ binding to F‐ATP synthase beta subunit triggers the mitochondrial permeability transition. EMBO Rep. 2017;18:1065–76. 10.15252/embr.201643354 28507163 PMC5494526

[8] Baines CP , Kaiser RA , Purcell NH , Blair NS , Osinska H , Hambleton MA , et al. Loss of cyclophilin D reveals a critical role for mitochondrial permeability transition in cell death. Nature. 2005;434:658–62. 10.1038/nature03434 15800627

[9] Palma E , Tiepolo T , Angelin A , Sabatelli P , Maraldi NM , Basso E , et al. Genetic ablation of cyclophilin D rescues mitochondrial defects and prevents muscle apoptosis in collagen VI myopathic mice. Hum Mol Genet. 2009;18:2024–31. 10.1093/hmg/ddp126 19293339

[10] Kirichok Y , Krapivinsky G , Clapham DE . The mitochondrial calcium uniporter is a highly selective ion channel. Nature. 2004;427:360–4. 10.1038/nature02246 14737170

[11] Santo‐Domingo J , Demaurex N . Calcium uptake mechanisms of mitochondria. Biochim Biophys Acta. 2010;1797:907–12. 10.1016/j.bbabio.2010.01.005 20079335

[12] Baughman JM , Perocchi F , Girgis HS , Plovanich M , Belcher‐Timme CA , Sancak Y , et al. Integrative genomics identifies MCU as an essential component of the mitochondrial calcium uniporter. Nature. 2011;476:341–5. 10.1038/nature10234 21685886 PMC3486726

[13] De Stefani D , Raffaello A , Teardo E , Szabo I , Rizzuto R . A forty‐kilodalton protein of the inner membrane is the mitochondrial calcium uniporter. Nature. 2011;476:336–40. 10.1038/nature10230 21685888 PMC4141877

[14] Perocchi F , Gohil VM , Girgis HS , Bao XR , McCombs JE , Palmer AE , et al. MICU1 encodes a mitochondrial EF hand protein required for Ca^2+^ uptake. Nature. 2010;467:291–6. 10.1038/nature09358 20693986 PMC2977980

[15] Plovanich M , Bogorad RL , Sancak Y , Kamer KJ , Strittmatter L , Li AA , et al. MICU2, a paralog of MICU1, resides within the mitochondrial uniporter complex to regulate calcium handling. PLoS One. 2013;8:e55785. 10.1371/journal.pone.0055785 23409044 PMC3567112

[16] Mallilankaraman K , Cardenas C , Doonan PJ , Chandramoorthy HC , Irrinki KM , Golenar T , et al. MCUR1 is an essential component of mitochondrial Ca^2+^ uptake that regulates cellular metabolism. Nat Cell Biol. 2012;14:1336–43. 10.1038/ncb2622 23178883 PMC3511605

[17] Raffaello A , De Stefani D , Sabbadin D , Teardo E , Merli E , Picard A , et al. The mitochondrial calcium uniporter is a multimer that can include a dominant‐negative pore‐forming subunit. EMBO J. 2013;32:2362–76. 10.1038/emboj.2013.157 23900286 PMC3771344

[18] Sancak Y , Markhard AL , Kitami T , Kovacs‐Bogdan E , Kamer KJ , Udeshi ND , et al. EMRE is an essential component of the mitochondrial calcium uniporter complex. Science. 2013;342:1379–82. 10.1126/science.1242993 24231807 PMC4091629

[19] Pallafacchina G , Zanin S , Rizzuto R . Recent advances in the molecular mechanism of mitochondrial calcium uptake. F1000Research. 2018;7:1858. 10.12688/f1000research.15723.1 PMC6263489 30555683

[20] Patron M , Granatiero V , Espino J , Rizzuto R , De Stefani D . MICU3 is a tissue‐specific enhancer of mitochondrial calcium uptake. Cell Death Differ. 2019;26:179–95. 10.1038/s41418-018-0113-8 29725115 PMC6124646

[21] Kamer KJ , Sancak Y , Fomina Y , Meisel JD , Chaudhuri D , Grabarek Z , et al. MICU1 imparts the mitochondrial uniporter with the ability to discriminate between Ca^2+^ and Mn^2+^ . Proc Natl Acad Sci USA. 2018;115:E7960–9. 10.1073/pnas.1807811115 30082385 PMC6112746

[22] Chaudhuri D , Artiga DJ , Abiria SA , Clapham DE . Mitochondrial calcium uniporter regulator 1 (MCUR1) regulates the calcium threshold for the mitochondrial permeability transition. Proc Natl Acad Sci USA. 2016;113:E1872–80. 10.1073/pnas.1602264113 26976564 PMC4822583

[23] Tomar D , Dong Z , Shanmughapriya S , Koch DA , Thomas T , Hoffman N , et al. MCUR1 Is a scaffold factor for the MCU complex function and promotes mitochondrial bioenergetics. Cell Rep. 2016;15:1673–85. 10.1016/j.celrep.2016.04.050 27184846 PMC4880542

[24] Kovacs‐Bogdan E , Sancak Y , Kamer KJ , Plovanich M , Jambhekar A , Huber RJ , et al. Reconstitution of the mitochondrial calcium uniporter in yeast. Proc Natl Acad Sci USA. 2014;111:8985–90. 10.1073/pnas.1400514111 24889638 PMC4066498

[25] Yamamoto T , Yamagoshi R , Harada K , Kawano M , Minami N , Ido Y , et al. Analysis of the structure and function of EMRE in a yeast expression system. Biochim Biophys Acta. 2016;1857:831–9. 10.1016/j.bbabio.2016.03.019 27001609

[26] Baradaran R , Wang C , Siliciano AF , Long SB . Cryo‐EM structures of fungal and metazoan mitochondrial calcium uniporters. Nature. 2018;559:580–4. 10.1038/s41586-018-0331-8 29995857 PMC6336196

[27] Fan C , Fan M , Orlando BJ , Fastman NM , Zhang J , Xu Y , et al. X‐ray and cryo‐EM structures of the mitochondrial calcium uniporter. Nature. 2018;559:575–9. 10.1038/s41586-018-0330-9 29995856 PMC6368340

[28] Nguyen NX , Armache JP , Lee C , Yang Y , Zeng W , Mootha VK , et al. Cryo‐EM structure of a fungal mitochondrial calcium uniporter. Nature. 2018;559:570–4. 10.1038/s41586-018-0333-6 29995855 PMC6063787

[29] Yoo J , Wu M , Yin Y , Herzik MA Jr , Lander GC , Lee SY . Cryo‐EM structure of a mitochondrial calcium uniporter. Science. 2018;361:506–11. 10.1126/science.aar4056 29954988 PMC6155975

[30] Wang Y , Nguyen NX , She J , Zeng W , Yang Y , Bai XC , et al. Structural mechanism of EMRE‐dependent gating of the human mitochondrial calcium uniporter. Cell. 2019;177(5):1252–61.e13. 10.1016/j.cell.2019.03.050 31080062 PMC6597010

[31] Tsai MF , Phillips CB , Ranaghan M , Tsai CW , Wu Y , Willliams C , et al. Dual functions of a small regulatory subunit in the mitochondrial calcium uniporter complex. Elife. 2016;5:e15545. 10.7554/eLife.15545 27099988 PMC4892889

[32] Yamamoto T , Ozono M , Watanabe A , Maeda K , Nara A , Hashida M , et al. Functional analysis of coiled‐coil domains of MCU in mitochondrial calcium uptake. Biochim Biophys Acta Bioenerg. 2019;1860:148061. 10.1016/j.bbabio.2019.148061 31394096

[33] Kitazawa M , Kubo Y , Nakajo K . The stoichiometry and biophysical properties of the Kv4 potassium channel complex with K^+^ channel‐interacting protein (KChIP) subunits are variable, depending on the relative expression level. J Biol Chem. 2014;289:17597–609. 10.1074/jbc.M114.563452 24811166 PMC4067195

[34] Yamada A , Yamamoto T , Yoshimura Y , Gouda S , Kawashima S , Yamazaki N , et al. Ca^2+^‐induced permeability transition can be observed even in yeast mitochondria under optimized experimental conditions. Biochim Biophys Acta. 2009;1787:1486–91. 10.1016/j.bbabio.2009.07.001 19616504

[35] Schmitt S , Saathoff F , Meissner L , Schropp EM , Lichtmannegger J , Schulz S , et al. A semi‐automated method for isolating functionally intact mitochondria from cultured cells and tissue biopsies. Anal Biochem. 2013;443:66–74. 10.1016/j.ab.2013.08.007 23969012

[36] Ran FA , Hsu PD , Wright J , Agarwala V , Scott DA , Zhang F . Genome engineering using the CRISPR‐Cas9 system. Nat Protoc. 2013;8:2281–308. 10.1038/nprot.2013.143 24157548 PMC3969860

[37] Yamamoto T , Yamada A , Watanabe M , Yoshimura Y , Yamazaki N , Yoshimura Y , et al. VDAC1, having a shorter N‐terminus than VDAC2 but showing the same migration in an SDS‐polyacrylamide gel, is the predominant form expressed in mitochondria of various tissues. J Proteome Res. 2006;5:3336–44. 10.1021/pr060291w 17137335

[38] Yamada A , Yamamoto T , Yamazaki N , Yamashita K , Kataoka M , Nagata T , et al. Differential permeabilization effects of Ca^2+^ and valinomycin on the inner and outer mitochondrial membranes as revealed by proteomics analysis of proteins released from mitochondria. Mol Cell Proteomics. 2009;8:1265–77. 10.1074/mcp.M800377-MCP200 19218587 PMC2690487

[39] Konig T , Troder SE , Bakka K , Korwitz A , Richter‐Dennerlein R , Lampe PA , et al. The m‐AAA protease associated with neurodegeneration limits MCU activity in mitochondria. Mol Cell. 2016;64:148–62. 10.1016/j.molcel.2016.08.020 27642048

[40] Liu JC , Liu J , Holmstrom KM , Menazza S , Parks RJ , Fergusson MM , et al. MICU1 serves as a molecular gatekeeper to prevent in vivo mitochondrial calcium overload. Cell Rep. 2016;16:1561–73. 10.1016/j.celrep.2016.07.011 27477272 PMC5316484

[41] Lee Y , Min CK , Kim TG , Song HK , Lim Y , Kim D , et al. Structure and function of the N‐terminal domain of the human mitochondrial calcium uniporter. EMBO Rep. 2015;16:1318–33. 10.15252/embr.201540436 26341627 PMC4662854

[42] Lee Y , Park J , Lee G , Yoon S , Min CK , Kim TG , et al. S92 phosphorylation induces structural changes in the N‐terminus domain of human mitochondrial calcium uniporter. Sci Rep. 2020;10:9131. 10.1038/s41598-020-65994-y 32499574 PMC7272466

[43] Ying WL , Emerson J , Clarke MJ , Sanadi DR . Inhibition of mitochondrial calcium ion transport by an oxo‐bridged dinuclear ruthenium ammine complex. Biochemistry. 1991;30:4949–52. 10.1021/bi00234a016 2036363

[44] Fieni F , Lee SB , Jan YN , Kirichok Y . Activity of the mitochondrial calcium uniporter varies greatly between tissues. Nat Commun. 2012;3:1317. 10.1038/ncomms2325 23271651 PMC3818247

[45] Payne R , Li C , Foskett JK . Variable assembly of EMRE and MCU creates functional channels with distinct gatekeeping profiles. iScience. 2020;23(4):101037. 10.1016/j.isci.2020.101037 32315830 PMC7170992

[46] Fan M , Zhang J , Tsai CW , Orlando BJ , Rodriguez M , Xu Y , et al. Structure and mechanism of the mitochondrial Ca^2+^ uniporter holocomplex. Nature. 2020;582:129–33. 10.1038/s41586-020-2309-6 32494073 PMC7544431

[47] Liu JC , Syder NC , Ghorashi NS , Willingham TB , Parks RJ , Sun J , et al. EMRE is essential for mitochondrial calcium uniporter activity in a mouse model. JCI Insight. 2020;5:e134063. 10.1172/jci.insight.134063 PMC7101141 32017711

[48] Chen L , Sun Q , Zhou D , Song W , Yang Q , Ju B , et al. HINT2 triggers mitochondrial Ca^2+^ influx by regulating the mitochondrial Ca^2+^ uniporter (MCU) complex and enhances gemcitabine apoptotic effect in pancreatic cancer. Cancer Lett. 2017;411:106–16. 10.1016/j.canlet.2017.09.020 28947137

