# Supplementary material for: Quantitative analysis of mitochondrial calcium uniporter (MCU) and essential MCU regulator (EMRE) in mitochondria from mouse tissues and HeLa cells
Source: FEBS Open Bio. 2022 Feb 23;12(4):811–26. doi: 10.1002/2211-5463.13371 (PMC8972046; doi:10.1002/2211-5463.13371)
Supplement: Supplementary file 1 — Fig. S1. Alignment of amino acid sequence of human MCU and mouse MCU; Q8NE86 and Q3UMR5, respectively. Asterisks show amino acids conserved between the 2 MCUs; and dots, similar amino acids. The epitopes of anti‐MCU antibodies are shown in red and by underbars. Fig. S2. Alignment of amino acid sequence of human EMRE and mouse EMRE; Q9H4I9 and Q9DB10, respectively. Asterisks show amino acids conserved between the 2 EMREs; and dots, similar amino acids. The epitopes of anti‐EMRE antibodies are shown in blue and by underbars. Fig. S3. Peptide mapping of the prepared proteins of MCU and EMRE. (A), The peptides identified by MS analysis were shown by black boxes in mouse MCU protein sequence: (B), the identified peptides were shown in EMRE protein sequences. The brief procedure of MS analysis is the followed. The prepared proteins of MCU and EMRE were purified by methanol/chloroform precipitation. The protein pellets were solubilized in 8 M urea followed by reduction in 2.5 mM DTT for 30 min at 37 °C, and alkylation in 55 mM iodoacetamide for 30 min at room temperature in the dark. After reducing the urea concentration to 1 M using 50 mM Tris‐HCl (pH 8.0), the proteins were digested at 37 °C overnight using 100 ng of trypsin/Lys‐C mix, Mass Spec Grade (Promega, Madison, WI). The prepared peptides were desalted with GL‐Tip SDB (GL Sciences, Tokyo, Japan), and the eluates were concentrated using a SpeedVac concentrator (Thermo Fisher Scientific). The prepared peptide solutions were dissolved with 0.1% TFA. LC‐MS/MS analysis of the prepared peptides was carried out on an EASY‐nLC 1200 UHPLC connected to a Q Exactive Plus mass spectrometer (Thermo Fisher Scientific). The peptides were separated on a 75‐μm inner diameter × 120‐mm C18 reversed‐phase column (Nikkyo Technos, Tokyo, Japan) using a linear gradient from 5 to 40% acetonitrile for 0‐60 min. A data‐dependent acquisition mode was used as the operation program of the mass spectrometer. Raw data were analyzed using the U [file FEB4-12-811-s004.docx]

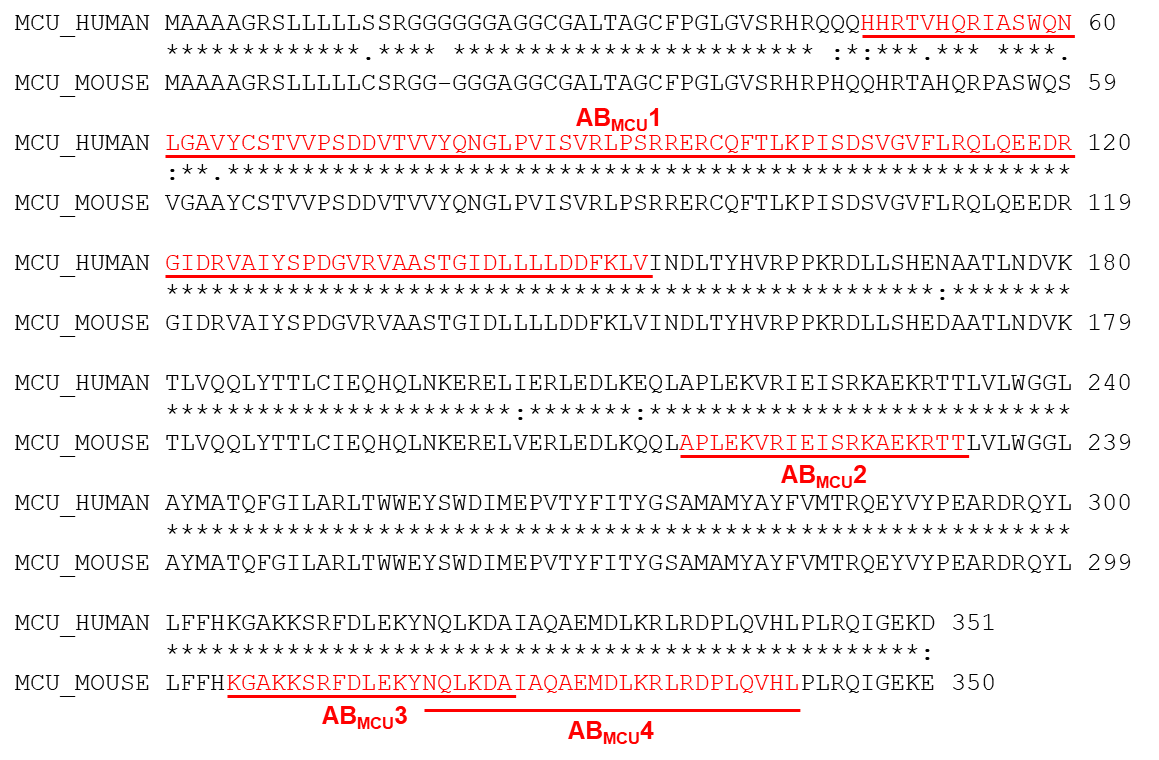


**Fig. S1.** Alignment of amino acid sequence of human MCU and mouse MCU; Q8NE86 and Q3UMR5, respectively. Asterisks show amino acids conserved between the 2 MCUs; and dots, similar amino acids. The epitopes of anti-MCU antibodies are shown in red and by underbars.


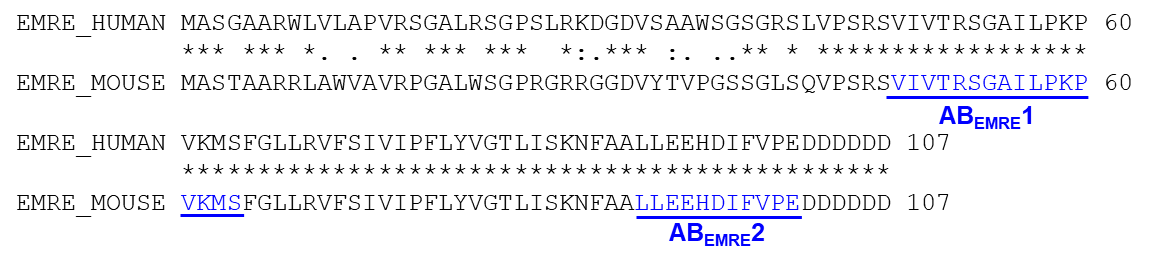


**Fig. S2.** Alignment of amino acid sequence of human EMRE and mouse EMRE; Q9H4I9 and Q9DB10, respectively. Asterisks show amino acids conserved between the 2 EMREs; and dots, similar amino acids. The epitopes of anti-EMRE antibodies are shown in blue and by underbars.


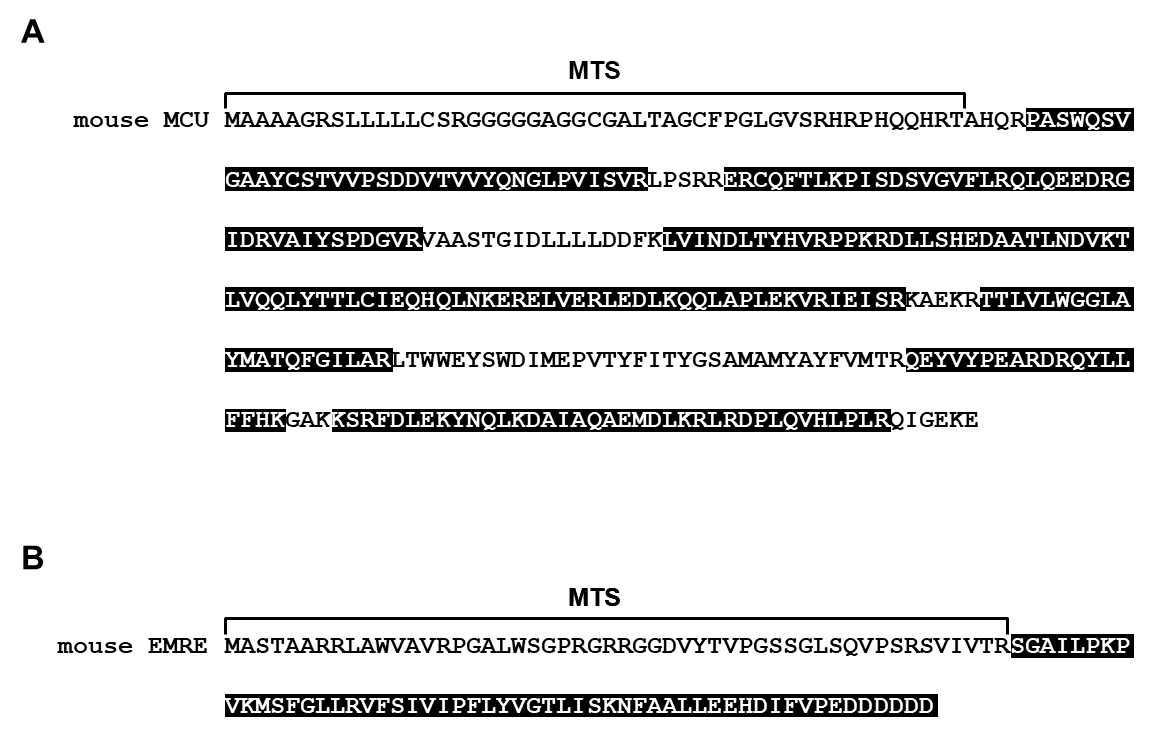


**Fig.S3** Peptide mapping of the prepared proteins of MCU and EMRE. **(A)**, The peptides identified by MS analysis were shown by black boxes in mouse MCU protein sequence: **(B)**, the identified peptides were shown in EMRE protein sequences. The brief procedure of MS analysis is the followed. The prepared proteins of MCU and EMRE were purified by methanol/chloroform precipitation. The protein pellets were solubilized in 8 M urea followed by reduction in 2.5 mM DTT for 30 min at 37 °C, and alkylation in 55 mM iodoacetamide for 30 min at room temperature in the dark. After reducing the urea concentration to 1 M using 50 mM Tris-HCl (pH 8.0), the proteins were digested at 37 °C overnight using 100 ng of trypsin/Lys-C mix, Mass Spec Grade (Promega, Madison, WI). The prepared peptides were desalted with GL-Tip SDB (GL Sciences, Tokyo, Japan), and the eluates were concentrated using a SpeedVac concentrator (Thermo Fisher Scientific). The prepared peptide solutions were dissolved with 0.1% TFA. LC-MS/MS analysis of the prepared peptides was carried out on an EASY-nLC 1200 UHPLC connected to a Q Exactive Plus mass spectrometer (Thermo Fisher Scientific). The peptides were separated on a 75-μm inner diameter × 120-mm C18 reversed-phase column (Nikkyo Technos, Tokyo, Japan) using a linear gradient from 5 to 40% acetonitrile for 0-60 min. A data-dependent acquisition mode was used as the operation program of the mass spectrometer. Raw data were analyzed using the UniProt database of Mus musculus with Proteome Discoverer, version 2.2 (Thermo Fisher Scientific) for peptide identification. The detail of the identified peptide were shown in Supplemental Table SII for recombinant MCU and Supplemental Table SIII for synthesized EMRE.

**Fig.S4.** Quantification analysis of MCU in the mitochondria by using anti-MCU antibody, AB_MCU_4. **A**, Schematic representation of epitopes of antibodies against mouse MCU; AB_MCU_1 and AB_MCU_4: the location of each epitope is shown by the bold line. **B**,**C**, **upper**, recombinant mature MCU protein (prepared in Figure 3) and the mitochondria isolated from mouse liver (**B**) and kidney (**C**) were subjected to SDS-PAGE followed by immunoblotting using AB_MCU_4; **lower**, The signal intensity of each band of recombinant mature MCU was detected by Image J; those signal intensities were plotted, resulting in calibration curves. The calculation of the amount of MCU protein from the signal intensities was carried out as shown in the legend of Fig 4. This amount was obtained by dividing the amount of MCU protein by the molecular weight (34843.02) of the mature MCU. The amount of MCU (fmol/µg) of obtained by using AB_MCU_4 (shown in light blue in the histogram) was compared with that obtained with AB_MCU_1 (shown in blue histogram) and shows that the amounts of MCU in liver mitochondria and kidney were almost the same.


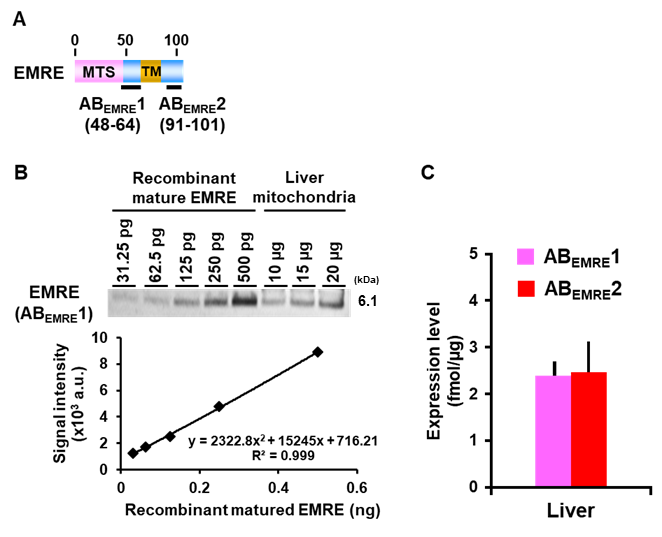


**Fig. S5.** Quantification analysis of EMRE in the mitochondria by using anti-EMRE antibody, AB_EMRE_1. **A**, Schematic representation of epitopes of antibodies against mouse EMRE; AB_EMRE_1 and AB_EMRE_2: the location of each epitope is shown by the bold line. **B**, **upper**, the recombinant mature EMRE (prepared in Figure 3) and the mitochondria isolated from mouse liver were subjected to SDS-PAGE followed by immunoblotting using AB_EMRE_1; **lower**, The signal intensity of each band of recombinant mature EMRE was detected by Image J; and those signal intensities were plotted, resulting in calibration curves. The calculation of the amount of EMRE protein from the signal intensities was carried out as shown in the legend of Fig 5. The averages of the calculated mitochondrial amount are shown in the histogram (**C**; mean ±s.d.), n≥3). **C**, The amount of EMRE (fmol) in 1 μg of the isolated mitochondria. This amount was obtained by dividing the amount of EMRE protein by the molecular weight (6109.04) of mature EMRE. The amount of EMRE (fmol/µg) obtained with AB_EMRE_1 (shown by the pink bar of the histogram) was compared with that using AB_EMRE_2 (shown by the red bar) and shows that the amounts of EMRE in liver mitochondria were almost the same with both antibodies.
